# Phlebitis Following the Hypodermic Use of Ergot in the Treatment of a Fibroid Tumor of the Uterus

**Published:** 1875-07-01

**Authors:** E. P. Allen

**Affiliations:** Geneva


### PHLEBITIS FOLLOWING THE HYPODERMIC USE OF ERGOT IN THE TREATMENT OF A FIBROID TUMOR OF THE UTERUS.

By E. P. Allen, M.D., Geneva.

ON the sixth day of November, A. D. 1871, I was consulted by Miss M. C. W., relative to a tumor that was giving her some trouble and a good deal of anxiety, situated in the pelvis, and had mounted up above the pubes.

She was forty-two years of age, and had had very good health up to a year and a half since, when she observed that her menses were very profuse and continued a day or two longer than formerly, and she felt weak and a dragging sensation through her hips and thighs that gave her a good deal of fatigue on walking.

She had suffered from dysmenorrhœa more or less since the menses first made their appearance. She was a school teacher from sixteen years of age up to thirty, and had worked at dress-making from that time to the present.

On making an examination, I found a firm tumor that mounted above and behind the pubes, of the size of a ball that is used in the game of base ball, and nearly as hard to the touch. The tumor completely filled the pelvis, resting upon the perineum, so it was with some difficulty that I passed my finger beneath and behind it. I was anxious to find the os uteri, and after a patient examination it was detected behind the pubes, and as high as I could reach with my finger, which only touched the lowest part of the os. I succeeded in passing into the uterus a flexible sound two and one-half inches. The fundus of the uterus had fallen back against the sacrum or rectum, and the os was drawn forward and upward to the extent aforesaid, with enlargement of the uterus above and below. I was satisfied the tumor was a fibroid of the uterus, and from its general appearance, belonged to the class known as subserous, or extra mural.

I had very little faith that any medicine would be of much benefit to her, but concluded to give her muriate of ammonia, as recommended by Dr. W. L. Atlee, of Philadelphia, hoping that it might retard, if it did not arrest, the growth. She took ten grains of muriate of ammonia three times a day before meals, and three drops of Fowler's solution after meals. This was continued five or six months without apparent benefit, the tumor increasing in size rapidly, when her stomach refused to tolerate the medicine.

About this time some friend of the patient advised her to take an infusion of red clover, which was persevered in for two months, when she procured the fluid extract of red clover, which she took three months longer without benefit.

On the sixth of November, 1872, I gave her another careful examination and found the tumor had increased greatly in size, filling up the entire front of the abdomen, extending above the umbilicus three and one-half inches. Her girth around the most prominent part of the abdomen was thirty-six inches. Her menses lasted six or seven days, and were very profuse during the whole time. I determined then to use ergot, after Prof. Hildebrandt's method.

In the evening of the same day, I gave her a hypodermic injection of Squibb's fluid extract of ergot, using fifteen drops of the ergot diluted with the same amount of water. The injections were inserted into the cellular tissue, beneath and around the umbilicus, at various distances over the tumor. They were always followed by considerable smarting, with a little swelling of the parts where they were introduced, which lasted several days.

After one week the dose was increased to twenty drops, and glycerine was substituted for water to reduce the ergot to lessen the pain when introduced, but the patient could detect no benefit in the relief of pain by the use of glycerine, and water was again used.

The fourth day following the use of ergot, the menses made their appearance and lasted between three or four days, and were not profuse at any time. Twelve days after the first use of ergot, I measured her again and found her girth to be thirty-four inches.

The treatment was continued until she had had thirty injections hypodermically, when it had to be suspended on account of smart febrile symptoms and severe pain in the left side and hip. Her girth had shrunk to thirty-two inches. The tumor had settled so that it was apparent to her friends. Dresses were worn with comfort that had not been worn for months, and she could walk with far greater ease. The hopes of the patient, as well as my own, were sanguine of a complete cure. At first the pain and fever it was thought would be transitory. She was ordered a saline cathartic, to be followed with Dover's powder, and hot fomentations applied over the painful region; but the pain and fever increased, and the pain extended in two or three days down the right leg to the foot. The entire limb and hip at the end of a week were greatly swelled, and the fever continued high, her pulse ranging from one hundred and twenty to twenty-five per minute. The superficial veins of the lower left half of the abdomen, hip and leg could be felt like cords under the finger. Red lines could be seen over the most superficial veins, and the parts were very painful to the touch.

It was now apparent that I had to deal with a severe case of phlebitis. A question with me was what was the cause of the disease. The above symptoms followed close after the last injection of ergot, and the pain started from that point. I had never learned that the use of ergot had produced phlebitis, but I knew very well that several authors had declared that it produced dry gangrene when it had been used in bread that contained a large amount of ergot, but this seemed to require its continuous use for some time. But none of those authors attribute the gangrene to phlebitis. Dr. George B. Wood says: "So far as concerns the direct systemic impression on the male system, it seems to be one of nervous depression, affecting both the animal and organic functions, and of course involving the heart." The action of the heart being weak, the capillary circulation becomes suspended and hence the death of the parts.

I thought it possible, as the tumor subsided, it might have settled into the pelvis and produced pressure, causing embolism or irritation of some of the veins, which was followed by inflammation, but I was never satisfied with this explanation or idea.

The entire limb was swollen apparently to nearly bursting, and the pain was intense.

The treatment consisted in keeping the bowels open with saline cathartics during the inflammatory period. Pain was greatly relieved by one-half grains of morphine, used hypodermically twice in twenty-four hours, and sleep was produced by taking twenty grains of hydrate of chloral in the evening. The morphine gave ease, but without the use of chloral she could not sleep. Hot fomentations were kept constantly applied to the limb, enveloped with a warm flannel blanket.

The acute inflammatión lasted about three weeks. The limb was still greatly swelled, and the skin presented a shining, tense appearance. The morphine now was used but once a day. The hot fomentations were suspended, and the limb enveloped with warm, thick cotton batting, retained in its place by a flannel blanket.

The chloral was required two weeks longer to procure sleep. By this time the tenderness had subsided very considerably. The limb was now rubbed thoroughly twice a day with the volatile liniment, and kept warm with the cotton as before. The foot was very susceptible to cold, which made it necessary to keep a warm soap-stone constantly to it. Ten days after this the limb was bandaged with a roller bandage, from the toes to the body, and the liniment used twice a day. Quinine was given after the acute stage of inflammation had subsided, and continued until the appetite returned.

The swelling gradually subsided, but it was eight weeks from the commencement of the phlebitis before the patient could put her foot to the floor, and three months before she could walk without assistance.

It will be observed by the foregoing description that the symptoms did not differ from any severe case of phlebitis or phlegmasia dolens.

I was uncertain of its cause, but was of opinion that it was the ergot that produced it. The patient was very confident this was the cause, and was rather afraid to have it used again hypodermically.

I related the case to quite a number of physicians, among whom was Dr. Washington L. Atlee, of Philadelphia, but none of them had heard of a similar case, and were not certain that the ergot was the cause of the phlebitis, and most of those consulted recommended a repetition of it. Three months after the treatment ceased, the menses became as profuse as before, and it was evident that the tumor was enlarging again. She was advised to take one and one-half drachms of fluid extract of ergot three times a day. This seemed to check the growth of the tumor, and arrested the profuseness of the menses, but after using it three months the stomach refused to tolerate it. The medicine caused vomiting nearly every time she took it. She then returned to Fowler's solution and muriate of ammonia, and continued it three or four months, but without being benefited. During this time the tumor had increased in size, and the menses were as profuse as ever. For a few months she took no medicines, and the tumor grew rapidly, and finally, despairing of getting help from any other source, she consented to another trial of the hypodermic use of ergot.

The ergot was commenced on the evening of the 13th of February, A. D. 1874, after carefully taking the size of her girth over the largest part of the abdomen, which was found to be thirty-nine inches. The tumor had mounted up within one inch and one-half of the ensiform cartilage. Fifteen drops of a solution of Squibb's solid extract of ergot was used, each drop representing one grain of ergot. The injections were used every evening, and the dose was in a few days increased to twenty drops, and continued eight weeks. The injections were most used on the right half of the abdomen, because they produced less pain than on the left, and it was apparent that the venous circulation was not as good on the left side as on the right. This preparation of ergot caused less pain than the fluid extract, and the small tumors produced by the injections were also less. The size of the abdomen did not diminish as rapidly as before, yet at the end of two weeks her girth had diminished one inch, and continued to decrease one inch every two weeks until she had used the remedy eight weeks, when her girth was between thirty-four and thirty-five inches. The menses had again diminished to their natural time and amount. I then increased the strength of the solution of ergot so that one drop represented two grains of ergot, with the hope that it would operate more rapidly upon the tumor. This strength caused a good deal of pain. The right side becoming quite tender, I used an injection on the left side about two and one-half inches below the umbilicus, and about the same distance from the median line. This caused so much pain, to relieve which cold applications were applied over the parts to prevent inflammation if possible, but in two or three days it was apparent that an abscess would form, after which warm fomentations were used until pus was formed and suppuration took place. During this time the use of ergot was suspended, which was fifteen days, when it was again used in the strength as at first; but was continued but ten days when it was apparent from the pain and enlargement of the veins over the right and lower half of the abdomen and hip, that there was to be a repetition of phlebitis on the right side. Vigorous means were at once adopted, hoping the inflammation might be arrested. Saline cathartics were promptly used, followed by the free use of carbonate of potass., while hot fomentations were faithfully applied, but all that we could do did not arrest it. The inflammation soon extended down the limb to the foot, and I may say that this case was a fac-simile of the case on the other side nearly two years before, only a little less severe, and the swelling not quite so great, yet it took quite as long a time before the patient could walk as in the first case.

It is unnecessary to give a detailed account of the treatment at this time, and I will merely say that it differed little from that used in the first case. The pain was arrested by the hypodermic use of morphine, and sleep was obtained with hydrate of chloral. The urine was occasionally loaded with urates, and relieved with carbonate of potass. Liniments and bandaging as before after the acute stage had passed. The patient was able to walk about the middle of July last, when treatment ceased.

The last of October she consulted me again. She complained of great tightness and more or less pain through and over the region of the tumor, and profuseness of the menses. I attributed these symptoms to the distension of the muscles of the abdomen, caused by the increasing growth of the tumor, and advised her to have ergot used in the arm hypodermically, fearing a repetition of phlebitis if it was used over the abdomen.

A solution of Squibb's solid extract was used, of the strength of one grain to one drop; was inserted in the region of the deltoid muscle. Fifteen drops were used at a dose. It was inserted alternately in her arms once a day. It produced a good deal of smarting and some swelling, which was relieved by the application of a cloth wet with cold water. The treatment continued twelve days, when the patient expressed herself a good deal relieved of her distressing symptoms, when the treatment ceased. No measurements were taken this time, for it was understood that she was not to be under treatment but a few days.

The action of ergot in producing phlebitis is a new thing to me, never having read of such action before or since, and so far as I know the case is unique.

In giving the history of the case in question, it has been my object to give the details of the use of the ergot, more than minute details of the growth and appearance of the tumor.

I submit the case to my medical brethren, without comment relative to the action of the ergot in producing phlebitis.

I have used ergot hypodermically in the treatment of congestion of the uterus, with menorrhagia, and to aid in the expulsion of polypus of the uterus, and given by the mouth in numerous cases, but never had unpleasant results follow its use excepting in the case related. Several patients have complained that it produced a weakness of the action of the heart.

